# Repair of symptomatic non-union rib fractures: outcomes from a contemporary thoracic surgical series

**DOI:** 10.1186/1749-8090-10-S1-A205

**Published:** 2015-12-16

**Authors:** Janani S Reisenauer, Brian D Kim, Stephen D Cassivi, William W Cross, David S Morris, Henry J Schiller

**Affiliations:** 1 Department of Surgery, Mayo Clinic, Rochester, MN, 55905, USA

## Background/Introduction

Rib fracture nonunion represents failure of normal fracture healing. Although randomized controlled trials have demonstrated benefit to acute rib stabilization, the role of open reduction and internal fixation (ORIF) for symptomatic nonunion fractures is unknown and limited to case reports.

## Aims/Objectives

We review and report our recent consecutive series of ORIF for symptomatic nonunion rib fractures.

## Method

All consecutive patients who underwent rib stabilization for symptomatic nonunion between 2010 and 2014 were retrospectively reviewed. Indications included persistent fracture on imaging accompanied with pain. Outcomes were analysed on 1) radiographic criteria including postoperative chest X-ray at 2 weeks, and CT scans at 3 and 6 months and 2) patient symptoms.

## Results

Eight patients (6 men, 2 women) underwent non-union rib stabilization of 1 to 4 ribs during the study period. Median age was 56 years (range, 46-67 years). Mean BMI was 31.8 and median interval from index injury to rib fracture surgical repair was 14.5 months (range, 4-24 months). 75% of this cohort used tobacco chronically within the 3 years preceding repair. One patient underwent stabilization with ORIF alone and the remaining 7 patients underwent ORIF plus autologous bone grafting. There was no operative mortality. Median length of stay was 3.5 days (range, 1-7 days). Complications included 2 surgical site infections treated with surgery and 1 patient with pneumonia requiring antibiotics. At a mean follow up of 9.8 months (range 1-27), all patients reported symptomatic improvement. Radiographic healing was present in 100%.

## Discussion/Conclusion

Rib stabilization with bone grafting may be a successful alternative in the management of symptomatic non-union rib fractures. With increased experience with this thoracic surgical option, earlier intervention in select cases may permit more rapid symptom control and better outcomes.

